# Promoter variation affects binding affinity of the transcription factor MdWRKY20 to the *Cell Wall Invertase 1* gene and decreases fructose content in apple fruit

**DOI:** 10.1093/hr/uhaf330

**Published:** 2025-12-05

**Authors:** Zhengyang Wang, Chunlei Zhang, Nanxiang Yang, Jian Huang, Fengwang Ma, Mingjun Li

**Affiliations:** College of Forestry, Northwest A&F University, Yangling, Shaanxi 712100, China; State Key Laboratory of Crop Stress Biology for Arid Areas/Shaanxi Key Laboratory of Apple, College of Horticulture, Northwest A&F University, Yangling, Shaanxi 712100, China; State Key Laboratory of Crop Stress Biology for Arid Areas/Shaanxi Key Laboratory of Apple, College of Horticulture, Northwest A&F University, Yangling, Shaanxi 712100, China; College of Forestry, Northwest A&F University, Yangling, Shaanxi 712100, China; State Key Laboratory of Crop Stress Biology for Arid Areas/Shaanxi Key Laboratory of Apple, College of Horticulture, Northwest A&F University, Yangling, Shaanxi 712100, China; State Key Laboratory of Crop Stress Biology for Arid Areas/Shaanxi Key Laboratory of Apple, College of Horticulture, Northwest A&F University, Yangling, Shaanxi 712100, China

## Abstract

Apple sweetness is primarily attributed to the high content and perceived sweet taste of fructose. A previous study used an F1 hybrid population of *Malus × domestica* [‘Honeycrisp’ (HC) × ‘Qinguan’ (QG) (2*n* = 34)] to identify quantitative trait loci (QTLs) for fructose content in fruit, revealing a stable QTL on linkage group (LG) 03 in the HC genetic map. In this study, gene ontology (GO) and **kyoto encyclopedia of genes and genomes (KEGG)** analyses of genes within this interval in combination with RNA-sequencing identified a cell wall invertase gene *MdCWINV1*, whose expression was highly associated with the dynamic changes in fructose content in parental fruits. The coding sequences were conserved between the two cultivars, while the promoters carried 73 single nucleotide polymorphisms (SNPs). Based on transcriptional regulatory element prediction, a unique SNP, CWINV1pro-1080 (A/C), located at −1080 bp upstream of the ATG start codon in the HC-P1 haplotype, was identified and predicted to affect the binding of the transcription factor MdWRKY20. β-glucuronidase (GUS) assays, chromatin immunoprecipitation-quantitative polymerase chain reaction (ChIP-qPCR), dual-luciferase assays, and genetic transformation confirmed that MdWRKY20 specifically binds to the CWINV1pro-1080 (A) haplotype and significantly suppresses *MdCWINV1* expression, reduces CWINV activity, and consequently decreases fructose accumulation. This study elucidated the functional role of *MdCWINV1* as a key gene regulating fructose content and clarified how natural mutations in its promoter influence gene expression and sugar composition.

## Introduction

Apple (*Malus* × *domestica* Borkh.) is one of the most economically valuable temperate fruit trees. The flavor quality of apple fruit is mainly influenced by the sugar, organic acid, and aromatic compound profiles and directly affects consumer acceptance. Among the flavor qualities, perceived sweetness reflects the content and composition of soluble sugars [1, 2], which are highly determined by genes related to sugar transport and metabolism [3, 4]. The main soluble sugars in mature apple fruits are fructose (Fru), sucrose (Suc), glucose (Glc), and sorbitol (Sor) [5]. The different sugars exhibit significant differences in sweetness, with Fru being the sweetest, followed by Suc, Glc, and Sor [6]. Therefore, elucidation of the regulatory mechanism determining Fru content in apple fruits is a key step in improving the quality of apple fruits through selective breeding.

Sugar transport and metabolism are critical processes in fruit development, influencing all stages from cell expansion to maturation. In apples, photosynthetic products produced by source organs (such as mature leaves) are transported through the phloem mainly as Sor and Suc to sink organs (such as fruits and young leaves). In young fruits, sugars transported through the phloem are unloaded into parenchyma cells along the concentration gradient through the symplastic pathway via plasmodesmata. As fruits mature and accumulate sugars, two other pathways are used. Sugars can be unloaded from the sieve element–companion cell complex (SE-CC) into the apoplastic space via transmembrane transport and then transported into the cytoplasm against the concentration gradient by Suc transporters (SUT) and Sor transporters (SOT) on parenchyma cells. Alternatively, Suc can first be hydrolyzed into hexoses by cell wall invertases (CWINVs) in the apoplast before transport into the cytoplasm via hexose transporters (HTs) [7, 8].

The accumulation of hexoses in fruits is closely associated with invertases (INVs) [9]. Plants possess three types of INVs: CWINV, vacuolar invertase (VINV), and cytosolic neutral invertase (NINV) [10]. Among them, CWINVs hydrolyze Suc into Fru and Glc and are highly expressed at the cell walls of sink organs [11]. As the Suc is unloaded from SE-CCs, the CWINVs reduce the Suc content in the apoplastic space to maintain the concentration gradient across the membrane, preventing the Suc from being reabsorbed by SUT on the SE-CC plasma membrane. The Suc-derived hexoses are then moved into sink organs [12]. This reduces unnecessary retransport of the Suc that enters the apoplast from either SE-CC or leaked from sink organs.

CWINVs have been extensively studied, especially in fruit and seed development [9]. Overexpression of *OsGIF1* (Grain Incomplete Filling 1) facilitates unloading of Suc in the endosperm of grains, influencing grain size and yield [13]. In tomatoes, silencing *sllin5* significantly reduces Suc cleavage and alters the morphology of flowers and fruits, leading to fruit abortion [14]. In *Arabidopsis*, specific silencing of *atcwinv2* and *atcwinv4* by microRNA results in arrested ovule development, affecting seed number [15]. In *Manihot esculenta*, overexpressing *MeCWINV3* accelerates Suc hydrolysis in leaves, inhibits Suc transport to storage roots, and reduces root yield [16]. Heterologous expression of *MdHT2.2* in tomatoes significantly increase *SlLIN5* expression and CWINV enzyme activity, while Suc content in fruits decreases significantly and hexose contents increase [17]. This indicates that *CWINV* somehow coordinates with transporters to regulate sugar distribution between source and sink organs, thereby influencing crop yield and quality.

Specific transcription factors are known to play crucial roles in sensing stress signals, responding to the expression of stress-responsive genes, and transmitting stress signals. Likewise, many transcription factors have been confirmed to regulate fruit sugar metabolism. For instance, the transcription factor ClNAC (NAM-ATAF-CUC-related gene) regulates Suc content in watermelon by inhibiting expression of *ClINV* [18]. In strawberry, the transcription factor FaMYB44.2 (v-myb avian myeloblastosis viral oncogene homolog) negatively regulates soluble sugar content by inhibiting expression of the sucrose-6-phosphate synthase *FaSPS3* (Sucrose Phosphate Synthase 3), with the interaction between FaMYB10 and FaMYB44.2 leadings to Suc accumulation in mature fruits [19]. In apples, many transcription factors involved in sugar metabolism have also been studied. The transcription factor MdAREB (ABA-responsive element-binding protein) directly activates *MdSUT2* expression, thereby increasing soluble sugar content in apple fruits [20]. MdDREB2A (dehydration-responsive element-binding protein) promotes soluble sugars accumulation by regulating vacuolar sugar transporters [21]. Sequence variation in the promoter of the sorbitol hydrogenase *MdSDH2* gene affects the binding ability of MdABI3 (Abscisic Acid Insensitive 3), thereby alters Fru content in apple fruits [22]. Under low-temperature treatment, MdCBF1/2 (C-repeat binding factor 1/2) regulate soluble sugar accumulation by affecting expression of the tonoplast sugar transporter genes *MdTST1* (Tonoplast Sugar Transporter 1) and *MdTST2* in apple fruits [23]. These studies demonstrate that multiple transcription factors participate in the regulation of genes related to sugar content, thereby influencing sugar accumulation in fruits.

This study further explored a previously identified apple QTL for Fru content [22] by tracking gene expression and sugar content during development of two apple cultivars. This study identified a candidate gene, *MdCWINV1*, which exhibited expression highly consistent with Fru content in the parental fruits. After promoter prediction and transcriptional regulation analysis, natural variations in the *MdCWINV1* promoter were found to affect the binding affinity ability of the transcription factor MdWRKY20. The altered binding influenced *MdCWINV1* expression and invertase activity, ultimately affecting sugar content in the fruits. This study aimed to identify genes regulating apple Fru content and to clarify the regulatory mechanism underlying variation in Fru content and has successfully provided potential genetic resources and markers for apple fruit quality improvement and molecular breeding.

## Results

### Bioinformatic analysis and candidate gene selection

**Figure 1:**
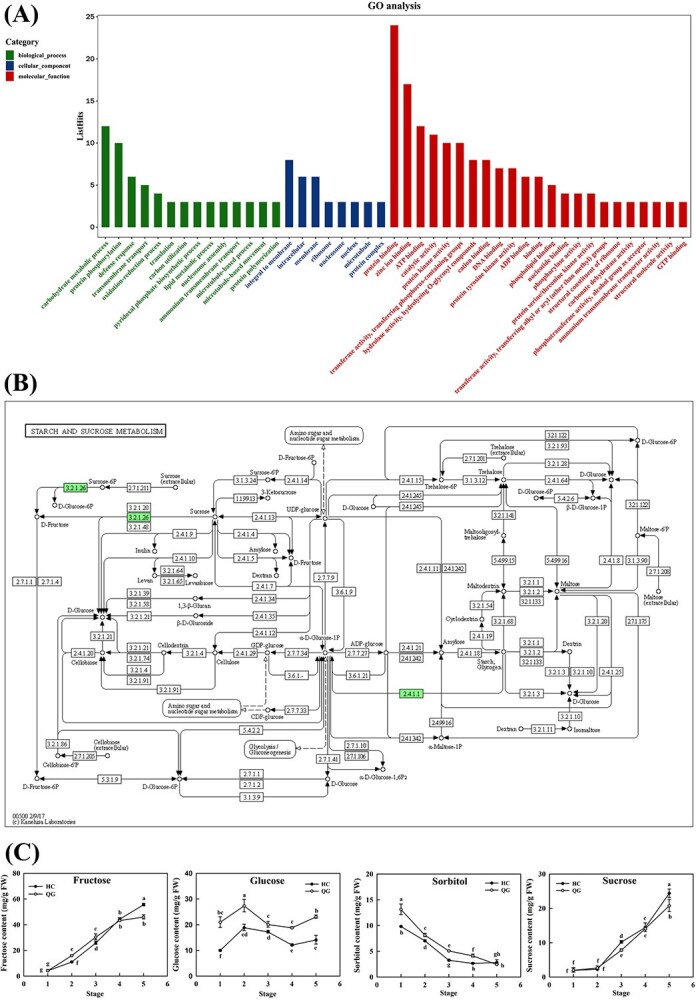
GO and KEGG analysis of the 219 candidate genes on QTL-Fru-LG03 in HC. (A) GO annotation of the 219 genes. *y*-axis indicates the number of genes within a GO term, *x*-axis is the GO term, the left ten belongs to the biological process category (green bar), the middle eight belongs to the cellular component category (blue bar), and the 23 on the right belong to the molecular function category (red bar). (B) KEGG pathway analysis of the starch and sucrose metabolism pathway. The three blocks (green blocks) different from others (transparent blocks) indicate the candidate gene families, namely 2.4.1.1 for glycogen phosphorylase and 3.2.1.26 for beta-fructofuranosidase. The analysis was performed in KAAS (https://www.genome.jp/tools/kaas/). (C) Changes in the contents of four sugar molecules in five stages of developing fruit from HC and QG. The *x*-axis indicates the five periods of fruit development: the cell division stage of young fruit (1), the early stage of cell expansion (2), the middle stage of cell expansion (3), the last stage of cell expansion (4), and the mature stage (5). Bars represent the mean value ± SE (*n* ≥ 3). The data were analyzed using one-way ANOVA test. Different letters indicate significant differences at *P* ≤ 0.05.

A stable QTL for fructose (Fru) content was previously identified on LG03 (20.95–23.31 cM) in the ‘Honeycrisp’ (HC) genetic map using an F1 hybrid population [HC × ‘Qinguan’ (QG)] (Fig. S1A and B) [22]. Within this interval, 219 genes were retrieved from the Rosaceae Genome Database (GDR) (Table S1). Gene Ontology (GO) analysis revealed enrichment in carbohydrate metabolic processes (12 genes), integral membrane components (8 genes), and protein binding functions (24 genes) (Fig. 1A). KEGG pathway analysis identified four pathways represented by five sugar metabolism-related genes: glycolysis/gluconeogenesis (K01689: *MDP0000313605*), pentose and glucuronate interconversions (K01051: *MDP0000625174/MDP0000457011*), galactose metabolism (K01193: *MDP0000932091*), and starch and sucrose metabolism (K01193: *MDP0000932091*, K00688: *MDP0000266061*) (Fig. 1B). Analysis of transcriptomes derived from three developmental stages of the two parental fruit cultivars showed that 93 genes were differentially expressed among those 219 genes (Fig. S2). Among the 93 genes with differential expression, through GO and KEGG annotation analysis, 17 genes related to carbohydrate metabolism or transport were identified. Only the CWINV gene *MDP0000932091* was associated with fructose content. The expression pattern of this gene in the two cultivated varieties was similar to the trend of sugar content change, so it was selected as a candidate gene for further analysis (Fig. S2, Fig. 1C).

### Structure and functional analysis of *MdCWINV1*

**Figure 2:**
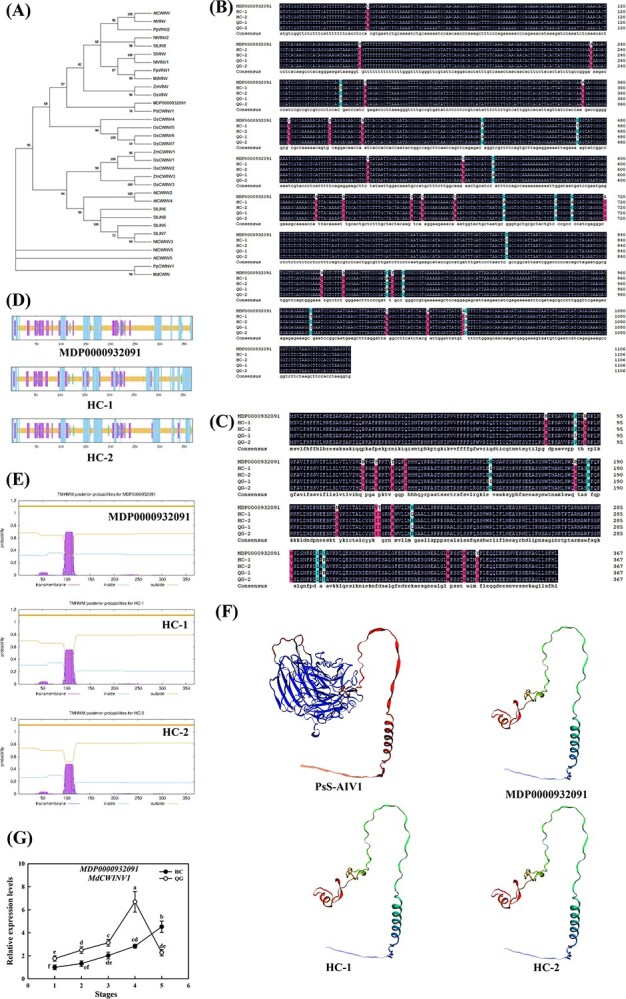
Phylogenetic evolutionary, sequence alignment, protein structure, transmembrane prediction, and transcription levels analysis of *MdCWINV1*. (A) Phylogenetic analysis of the MdCWINV1 amino acid sequence with INVs from *A. thaliana*, *Solanum lycopersicum*, *Zea mays*, *Nicotiana tabacum*, *Prunus persica*, *Malus domestica*, *Pyrus bretschneideri*, and *Oryza sativa*. (B) Alignment of the coding sequences of *MdCWINV1* in HC, QG, and ‘Golden Delicious’ (GD, reference sequence from GDR). (C) Alignment of the amino acid sequences of MdCWINV1 from HC, QG, and GD. (D) Protein secondary structure prediction of MdCWINV1 from HC, QG, and GD. (E) Transmembrane structure prediction of MdCWINV1 from HC, QG, and GD. (F) Tertiary structure prediction of MdCWINV1 from HC, QG, GD, and *P. pyrifolia*. (G) Expression of *MdCWINV1* in developing fruits of HC and QG. The expression in young fruit (stage 1) was set as 1. The numbers 1/2/3/4/5 represent five periods of fruit development: the cell division stage of young fruit (1), the early stage of cell expansion (2), the middle stage of cell expansion (3), the last stage of cell expansion (4), and the mature stage (5). Bars represent the mean value ± SE (*n* ≥ 3). The data were analyzed using one-way ANOVA test. Different letters indicate significant differences at *P* ≤ 0.05.

Phylogenetic analysis indicated that MDP0000932091 clustered closely with PbCWINV1 from pear (Fig. 2A), so it was designated *MdCWINV1*. Cloning of the *MdCWINV1* coding sequences from HC and QG (mRNA) revealed two haplotypes per cultivar (HC-1, HC-2; QG-1, QG-2), the total length of the coding sequence of this gene is 1107 bp, encoding 368 amino acids. Sequence alignment showed 12 SNPs leading to six amino acid changes, with HC-1 identical to QG-1 and HC-2 identical to QG-2 (Fig. 2B and C). Predictions of the physicochemical properties and secondary structures of the HC-1/QG-1 and HC-2/QG-2 proteins were highly similar, differing slightly at the C-termini (Table S2, Fig. 2D). All haplotypes contained a large predicted transmembrane domain (~100 aa) (Fig. 2E). Homology modeling of tertiary structures showed high consistency in the core region (68–199 aa, A0A7Y9_PYRPY model, gene: PsS-AIV1, organism: *Pyrus pyrifolia* var culta) (Fig. 2F), the other parts also have high similarity in secondary and tertiary structures. Prediction analysis indicates that there is no significant difference in the protein characteristics and functions, suggesting coding sequence variations were unlikely responsible for Fru content differences. The proteins encoded by HC-1 and HC-2 sequences exhibited high similarity. In subsequent experiments, HC-1 was selected as the research subject. Expression analysis during fruit development showed a continuous increase of *MdCWINV1* in HC, while in QG gene expression peaked mid-development and declined sharply thereafter. *MdCWINV1* expression was significantly decreased in QG than HC during early and midstages, until the expression level plummeted in QG at maturity (Fig. 2G).

**Figure 3:**
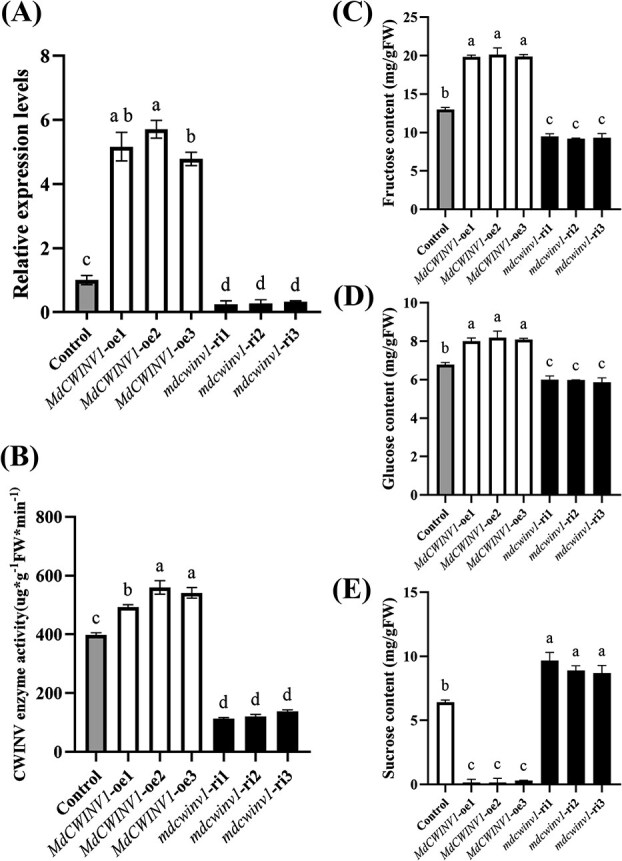
Expression levels, enzyme activity, and sugar content analysis of *MdCWINV1* transgenic lines. (A) qRT-PCR detection of *MdCWINV1* overexpression and RNAi transgenic lines. (B) CWINV enzyme activities of the *MdCWINV1* overexpression and RNAi transgenic lines. (C–E) Sugar contents of the *MdCWINV1* overexpression and RNAi transgenic lines. Bars represent the mean value ± SE (*n* ≥ 3). The data were analyzed using one-way ANOVA test. Different letters indicate significant differences at *P* ≤ 0.05.

To verify *MdCWINV1* function, overexpression and silencing vectors were transformed into calli derived from ‘Orin’ apple flesh. qRT-PCR analysis indicated that *MdCWINV1* was overexpressed or silenced in different lines (Fig. 3A). CWINV activities were significantly higher in overexpression lines (oe) and lower in silenced lines (ri) compared to the control (Fig. 3B). The overexpression lines contained ~50% higher Fru and ~20% higher Glc, with nearly depleted Suc levels compared to the control, while silencing lines had ~20% lower Fru, ~10% lower Glc, and ~25% higher Suc compared to the control (Fig. 3C–E). These results demonstrated that *MdCWINV1* hydrolyzes Suc, thereby reducing Suc levels and promoting hexose accumulation.

### Promoter variants and transcription factor binding affinity analysis

Given that no significant differences exist in the coding and amino acid sequences of *MdCWINV1* between HC and QG, it was hypothesized that the difference in Fru content between the two cultivars results from differential *MdCWINV1* expression. Transcriptome and qRT-PCR analyses of samples from these two cultivars during fruit development confirmed this. Cloning the ~2000-bp promoter regions identified four promoter haplotypes (HC-P1, HC-P2, QG-P1, QG-P2) with a total of 73 SNPs (35 between HC haplotypes, 55 between QG haplotypes) (Fig. S3A).

The four promoter haplotypes were analyzed against the Plant Transcriptional Regulatory Map and Plant *Cis*-Acting Regulatory Element (PlantCARE) databases. Among the four haplotypes, there were 26, 2, 38, and 49 predicted specific transcription factor binding sites, respectively (Tables S3 and S4). A W-box element (GGTCAA) predicted to specifically bind a WRKY transcription factor was uniquely present in HC-P1 at −1080 bp upstream of the ATG, whereas the corresponding sequence in other haplotypes was GGTCCA. The SNP site at this position was named CWINV1pro-1080 (A/C). Transcriptome and qRT-PCR analyses identified a highly expressed gene, *MDP0000184361*, during fruit development that belongs to the WRKY family. The expression of *MDP0000184361* was negatively associated with the dynamic changes in Fru content and with *MdCWINV1* expression in both cultivars (Fig. S3B and C). Phylogenetic analysis showed that MDP0000184361 was most closely related to AtWRKY20 in *Arabidopsis thaliana*, so it was named MdWRKY20 (Fig. S3D).

**Figure 4:**
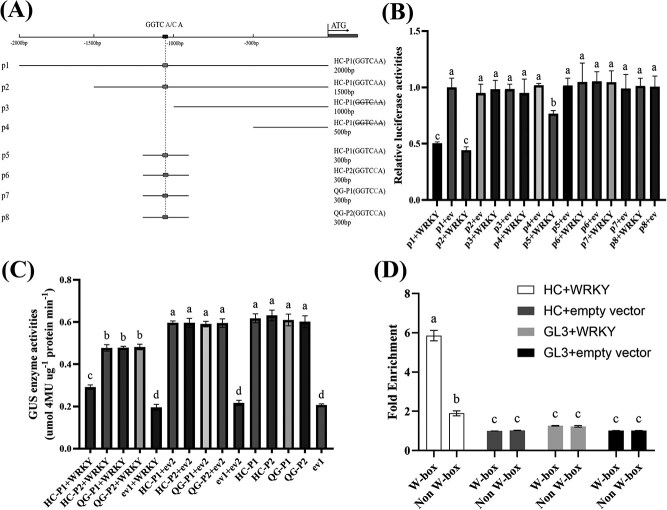
Transcriptional regulation of *MdCWINV1* promoter through interaction with MdWRKY20. (A) Promoter sequences used in vectors for luciferase assays. p1 and p2 both contain the HC-P1 SNP CWINV1pro-1080(A), with p1 representing the full-length promoter and p2 a 5′-truncated version, while p3 and p4 exclude the CWINV1pro-1080(A) SNP. p5, p6, p7, and p8 are 300-bp fragments of HC-P1/P2 and QG-P1/P2 around the SNP CWINV1pro-1080(A/C), respectively. (B) Analysis of luciferase activities in vectors carrying promoters of different length and haplotypes in the presence and absence of recombinant *MdWRKY20* vectors transiently transformed in tobacco leaves. (C) GUS activity in apple callus transiently coexpressing different combinations of *MdCWINV1pro:GUS* (HC-P1/P2, QG-P1/P2) and *35Spro:MdWRKY20*. ev1: empty vector1, pBI121; ev2: empty vector2, pCAMBIA-2300. (D) ChIP-qPCR assay of the interaction between MdWRKY20 and the different promoters of *MdCWINV1* in the leaves of apple cultivars HC and GL3 transiently transformed with CaMV35S*:MdWRKY20*-GFP. The nonbinding value was set to 1. Empty vector: pCAMBIA-2300. Bars represent the mean value ± SE (*n* ≥ 3). The data were analyzed using one-way ANOVA test. Different letters indicate significant differences at *P* ≤ 0.05.

The HC-P1 promoter was first truncated to determine the MdWRKY20-binding region, and then 300-bp fragments containing the CWINV1pro-1080 (A/C) site were amplified from HC-P1, HC-P2, QG-P1, and QG-P2 for analyzing the MdWRKY20-binding affinity for the promoter haplotypes (Fig. 4A). Dual-luciferase assays with truncated HC-P1 promoter fragments localized the functional binding site from −1500 to −1000 bp [p1, p2; containing CWINV1pro-1080(A)], consistent with the predicted site at −1080 bp (Fig. 4B). Assays with the 300-bp fragments containing CWINV1pro-1080(A/C) showed that MdWRKY20 suppressed only the luciferase activity when driven by the HC-P1 haplotype (GGTCAA), but not other haplotypes (GGTCCA) (Fig. 4B).

GUS assays showed that when the four haplotype promoters were transformed alone or cotransformed with the empty vector into ‘Orin’ fruit calli tissues, there was no significant difference in GUS activity among them. However, cotransfection of these promoters with *MdWRKY20*, significantly decreased GUS activity in all cases, with the HC-P1 showing significantly lower activity compared to the other three haplotypes (Fig. 4C).

The *MdWRKY20-oe* vector was transiently transformed into tissue-cultured shoots of HC and ‘Gala 3’ [GL3, CWINV1pro-1080(C), identical promoter sequence to QG] for ChIP-qPCR analysis. The ChIP-qPCR assays showed that the *MdCWINV1* promoter region containing the W-box element [CWINV1pro-1080(A)] in HC was significantly enriched in leaves transformed with MdWRKY20-GFP, while little to no enrichment was observed in non-W-box regions in HC or GL3 (Fig. 4D). These results indicate that MdWRKY20 can bind to the HC-P1 promoter sequence through the W-box element and negatively regulate *MdCWINV1* expression.

### Overexpression of *MdWRKY20* affects *MdCWINV1* expression, CWINV activity, and sugar contents

**Figure 5:**
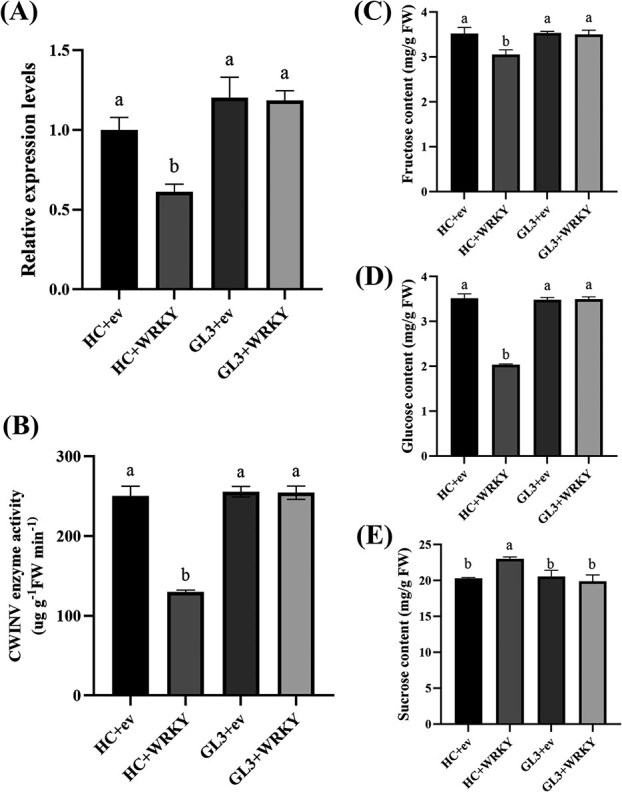
*MdCWINV1* expression, CWINV activity and sugar contents in HC and GL3 leaves with *MdWRKY20* transient transformation. (A) Transcript levels of *MdCWINV1*. *MdActin* (MD01G1001600) was used as an internal control. (B) Enzyme activities of CWINV in HC and GL3 leaves with empty vector or *MdWRKY20* transient transformation. (C–E) Fru, Glc, and Suc contents of transient transgenic lines. ev: empty vector, pCAMBIA-2300. Bars represent the mean value ± SE (*n* ≥ 3). The data were analyzed using one-way ANOVA test. Different letters indicate significant differences at *P* ≤ 0.05.

To verify if the binding of the transcription factor MdWRKY20 to the promoter of *MdCWINV1* affects sugar content, the *MdWRKY20* gene was overexpressed in leaves of the HC [CWINV1pro-1080(A)] and GL3 [CWINV1pro-1080(C)] cultivars. Overexpression of *MdWRKY20* led to a nearly 40% decrease in *MdCWINV1* expression in HC leaves compared to the control (Fig. 5A), a 50% decrease in CWINV activity (Fig. 5B), significant reductions in Fru and Glc contents, and a significant increase in Suc content. In contrast, no significant changes occurred in GL3 leaves carrying an empty vector or the *MdWRKY20* vector (Fig. 5C–E).

## Discussion

The sugar content of fruits is mainly influenced by sugar metabolism and transport. In most higher plants, Suc is both the primary photosynthetic product and the main carbohydrate transported over long distances from source to sink tissues. In fruits, there are two paths for uptake of Suc into the parenchyma cells (PCs): one is directly through Suc transporters (SUT/SUC) on the plasma membrane, and the other is through hydrolysis of Suc by CWINV to generate Fru and Glc, which are then taken in through HTs on the plasma membrane. CWINV catalyzes the irreversible hydrolysis of Suc into Fru and Glc and plays a crucial role in regulating sink strength and source–sink balance [24]. In sink organs, the main function of CWINV is to promote the unloading and utilization of Suc. CWINV reduces the concentration of Suc in the apoplastic space through hydrolysis, maintaining the concentration gradient between source and sink to ensure the continuous transport of photosynthetic products, and facilitates the transport of sugars from the apoplast into parenchyma cells [25].

In this study, genes within the major QTL for Fru content were identified and a key candidate gene, *MdCWINV1*, was selected based on its expression in apple fruit in a pattern consistent with the Fru contents in the parents. Through bioinformatics analysis of the four haplotype sequences of the *MdCWINV1* gene, it was found that there were no significant differences among them. This indicates that the minor differences in the coding sequence are unlikely to be the main cause of the variation in parental Fru content. However, this part still requires further verification. Genetic transformation confirmed that *MdCWINV1* significantly increased CWINV enzyme activity, hydrolyzing Suc and promoting Fru accumulation. Similar results were reported in species such as passion fruit [26], strawberry [27], pear [28], and grape [29], where altering CWINV gene expression in fruits significantly altered sugar content. These results indicate that in apple fruits, *MdCWINV1* is a key gene for the genetic regulation of Fru content.

Transcriptional regulation is the main mechanism controlling how genetic information encoded by genes is transformed into phenotypes. At the transcriptional level, the promoter plays a critical role. Natural promoter variations cause differences in transcriptional regulation, ultimately leading to trait variations and are an important genetic basis for intraspecific phenotypic diversity [30]. This study compared the apple cultivars HC and QG, which differ significantly in the content of Fru in their mature fruits, and showed that *MdCWINV1* expression was consistent with the variation in Fru content. The alignment analysis of the four promoter sequences indicated that there were 73 SNPs, a unique SNP CWINV1pro-1080 (A) formed the W-box element in HC-P1 that binds MdWRKY20 were identified, this is specifically present in a haplotype of HC and only influences HC-P1. While other 72 SNPs might also result in the alteration of *cis*-acting elements, thereby affecting the binding of transcription factors. It can occur by altering the affinity with these transcription factors, or the specific binding of other transcription factors, but this still requires further research.

*MdWRKY20* expression decreased in HC during fruit maturation, but decreased initially then increased in QG. MdWRKY20 could bind to the *MdCWINV1* HC-P1 promoter haplotype to inhibit its expression, which corresponded to the lower Fru content in HC in the early stage of fruit development. As the expression of *MdWRKY20* decreased in the later stage of fruit development, the inhibition on *MdCWINV1* weakened, leading to a significant increase in the expression of *MdCWINV1* and Fru content in HC. Whether this site can bind to other WRKY transcription factors to promote *MdCWINV1* expression in the later stage of fruit development still needs further analysis. Based on the upper analyses, no transcription factors that are highly expressed in the later stage and are highly correlated with the sugar content change pattern of the fruit have been screened out. It is speculated that the variation of other SNP sites in the promoter region of *MdCWINV1* may be regulated by other positive regulatory transcription factors, causing higher expression of HC-*MdCWINV1* than QG-*MdCWINV1* and higher fructose content in HC fruits than QG fruits in the later stages of fruit development, and this still needs further exploration.

The WRKY family is one of the largest transcription factor families in plants and participates in processes such as metabolic regulation, growth and development, and stress responses. Research has demonstrated that WRKY transcription factors can affect Suc accumulation in fruits. MdWRKY32 increases the expression of the key starch degradation gene *MdBAM5* by binding its promoter, thereby accelerating the conversion of starch to soluble sugar in postharvest apples [31]. The pitaya HpWRKY3 can directly activate the Suc metabolism genes *HpINV2* and *HpSuSy1*, increasing the Suc accumulation by 58% during fruit ripening [32]. Zhang *et al*. found that overexpression of *MdWRKY126* downregulated the expression of genes encoding enzymes such as CWINV and transporters such as HTs and TSTs, leading to a decrease in hexose levels in apple calli and tomato fruits [33], which is consistent with the results of our experiment. For fruit production, the CWINV1pro-1080 (A) haplotype has less advantage compared to the CWINV1pro-1080 (G) haplotype. According to the results, MdWRKY20 specifically binds to this site to negatively regulate the expression of *MdCWINV1*, thereby reducing the fructose content. This has a negative impact on fruit quality. Therefore, by developing this SNP into a molecular marker, it can be utilized for the selection of hybrid parents and the early trait screening of offspring, enabling molecular-assisted breeding and enhancing breeding efficiency.

**Figure 6:**
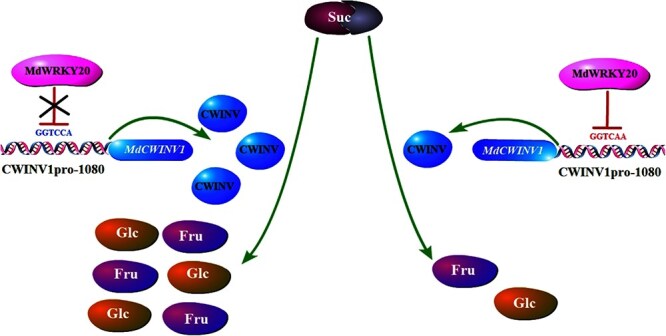
Natural SNP in the *MdCWINV1* promoter affects its transcription level by influencing the binding affinity of MdWRKY20, which leads to altered Fru content. The *MdCWINV1* gene encodes a CWINV, which catalyzes conversion of Suc into Fru. An SNP (A/C) in the *MdCWINV1* promoter region (SNP site CWINV1pro-1080) affected the binding affinity of the transcription factor MdWRKY20, which downregulates *MdCWINV1* expression level when bound to the promoter. Promoters with the A (right part, red letters) at SNP site CWINV1pro-1080 had a stronger binding affinity with MdWRKY20 than those with C (left part, blue letters), resulting in lower expression of *MdCWINV1*. As a result, a genotype with an A at CWINV1pro-1080 has lower Fru content than one with the C polymorphism.

Overall, this study identified *MdCWINV1* as the key gene underlying the Fru QTL located on the HC LG03. This gene encodes an invertase that cleaves Suc into hexoses, which can then be transported into fruit cells and serve to promote Fru accumulation. The expression of this gene showed significant differences between the parental lines and was consistent with the trends of Fru content. The natural variation identified in this promoter at SNP CWINV1pro-1080 (A/C) alters MdWRKY20-binding affinity, modulating *MdCWINV1* expression and, ultimately, Fru content (Fig. 6). This key gene *MdCWINV1* and its promoter SNP can be used for molecular breeding, early screening, and cultivar identification in apple plants.

## Materials and methods

### Plant materials

Fruit samples were collected from ‘Honeycrisp’ (2n = 34) (HC-1/2/3/4/5) at 15, 45, 75, 105, and 120 days after blooming (DAB) and from ‘Qinguan’ (2n = 34) (QG-1/2/3/4/5) at 15, 45, 90, 135, and 175 DAB. The numbers 1/2/3/4/5 represent five key fruit development stages: (i) the cell division stage of young fruit, (ii) the early stage of cell expansion, (iii) the middle stage of cell expansion, (iv) the last stage of cell expansion, and (v) the mature stage, respectively. Three biological replicates were established, with five fruits collected per replicate [22].

‘Orin’ apple calli were subcultured biweekly using the callus subculture medium under dark conditions in a culture room at 25°C with shaking at 150 rpm [22, 34]. HC and Gala 3 (GL3) tissue-cultured shoots were subcultured monthly on MS-based medium (4.43 g/l MS, 15 g/l sucrose, 8 g/l agar powder, 0.2 mg/l IAA, and 0.3 mg/l 6-BA) at 25°C under 16 h light/8 h dark photoperiod in a culture room [34].

### Measurement of sugar content and CWINV enzyme activity

Soluble sugars were extracted with 75% methanol, using ribitol as an internal standard. Samples were dried under vacuum, derivatized with methoxyamine hydrochloride and N-methyl-N-trimethylsilyl-trifluoroacetamide, and analyzed using a GCMS-2010 SE (Shimadzu Corporation, Tokyo, Japan) equipped with a DB-5 MS capillary column (20 m × 0.18 mm × 0.18 μm) [22].

CWINV activity was analyzed using the Cell Wall-Bound Acid Invertase (CWI) Activity Assay Kit (Boxi Biotechnology, Beijing, China). The crude enzyme extract was prepared following the manufacturer’s protocol. Briefly, 200 μl crude enzyme solution was mixed with 800 μl Reagent 1, mixed by vortex, and then incubated at 37°C for 30 min. Reagent 3 (800 μl) was added and mixed. Reactions were terminated in boiling water for 10 min, then cooled to room temperature. Absorbance was measured at 540 nm, and the enzyme activity was calculated.

### Candidate gene selection and annotation

The QTL region associated with Fru content on LG03 of HC was analyzed in a previous study [22]. For the RNA-seq data of HC and QG, it can be obtained at NCBI with the accession number PRJNA1344609 (https://www.ncbi.nlm.nih.gov/bioproject/PRJNA1344609/). A total of 219 genes were predicted within the locus. The chromosomal positions and functional annotations of these genes were retrieved from the Rosaceae Genome Database (Rosaceae GDR) (Malus × domestica Genome v1.0; http://www.rosaceae.org/species/Malus/malus_x_domestica/genome_v1.0) [35]. Functional classification and GO annotation were conducted using the WEGO 2.0 platform [36]. KEGG pathway analysis was performed via the KEGG Automatic Annotation Server (KAAS) [37].

### Phylogenetic and molecular analysis of candidate genes

The predicted amino acid sequences for the 219 genes were obtained from both the GDR and NCBI databases (http://www.ncbi.nlm.nih.gov). Phylogenetic trees were constructed using the maximum-likelihood method with MEGA X. Sequence alignment was performed using DNAMAN 8 (LynnonBiosoft, Vaudreuil-Dorion, Canada) with default parameters. *Cis*-acting regulatory elements in the promoter regions were predicted using the Plant *Cis*-Acting Regulatory Element database (PlantCARE, http://bioinformatics.psb.ugent.be/webtools/plantcare/html/) [38]. Transcription factor binding sites were analyzed using the Plant Transcriptional Regulatory Map database (https://plantregmap.gao-lab.org/binding_site_prediction.php) [39]. Physicochemical properties of the proteins were evaluated using ProtParam (Expasy). Secondary structure prediction was conducted using SOPMA (https://npsa-prabi.ibcp.fr/cgi-bin/npsa_automat.pl?page=/NPSA/npsa_sopma_f.html), transmembrane domains were identified using TMHMM-2.0 (https://services.healthtech.dtu.dk/services/TMHMM-2.0/), and tertiary structures were modeled through homology using SWISS-MODEL (https://swissmodel.expasy.org/).

### RNA extraction and qRT-PCR analysis

Total RNA was isolated with the RNAprep Plant Kit (Tiangen, Beijing, China) according to the manufacturer’s instructions. RNA purity and concentration were assessed by agarose gel electrophoresis and NanoDrop 2000 spectrophotometer, respectively. First-strand cDNA was synthesized with the PrimeScript RT Kit (TAKARA, Dalian, China). Gene-specific primers were designed based on available sequences from the NCBI database (https://www.ncbi.nlm.nih.gov/) and are listed in Table S5. Primer specificity was confirmed via primer-BLAST analysis on NCBI and melt curve analysis during qRT-PCR. qRT-PCR was performed using the Gentier 96E system (TIANLONG, Xi’an, China). Each sample was analyzed in triplicate. The expressions of the target genes in apples were normalized to *MdActin* (MD01G1001600) as the internal control.

### Plasmid construction

Vector construction was performed according to Wang *et al*. [22]. The haplotypes of full-length coding sequences (HC-1/2, QG-1/2) and promoter sequences (2000-bp promoter regions upstream of the ATG codon, HC-P1/P2, QG-P1/P2) of the candidate gene *MdCWINV1* were cloned from HC and QG fruit sample and reorganized into pMD19-T vector (Takara, Dalian, China) for sequencing. After sequencing, the full-length coding sequence and an interference-targeting fragment from HC-1 were cloned and inserted into the pCAMBIA-2300 vector and the pTRV2 vector, respectively, via PCR, to construct the pCAMBIA-2300-*MdCWINV1* recombinant plasmid as the overexpression vector and the pTRV2-*MdCWINV1* recombinant plasmid as the RNA interference vector (RNAi, RNA-interference). Each construct was transformed into calli tissue derived from ‘Orin’ fruit flesh through *Agrobacterium*-mediated transient transformation.

The HC-P1/2 and QG-P1/2 promoter sequences obtained through screening were cloned into the pBI121 vector (containing the CaMV 35S promoter and the GUS reporter gene) forming the pBI121-MdCWINV1pro recombinant plasmid. Additionally, different fragments of the HC-P1 promoter haplotype (2000, 1500, 1000, and 500 bp in length, labeled as p1, p2, p3, and p4, respectively) were cloned and inserted into the pGreenII0800-luc vector to generate the pGreenII0800-p1/p2/p3/p4-luc recombinant plasmids. Both p1 and p2 contain the SNP CWINV1pro-1080(A), while p3 and p4 do not. p5/p6/p7/p8 are 300-bp fragments derived from the HC-P1, HC-P2, QG-P1, and QG-P2 haplotypes and surrounded the CWINV1pro-1080(A/C) SNP. The full-length coding sequence of *MdWRKY20* was cloned into the pCAMBIA-2300 and pGreenII 62SK vectors to form the pCAMBIA-2300-*MdWRKY20* and pGreenII 62SK-*MdWRKY20* recombinant plasmids.

### Plant transformation

The transient transformation of ‘Orin’ fruit pulp callus was performed according to Wang *et al*. [22]. Callus tissues were cultured in liquid medium at 25°C and 120 rpm in the dark for 10–12 days and kept ready for use. Callus tissue was collected through a sterile 200-mesh nylon net, infected in the *Agrobacterium* resuspension solution for 15 min, transferred onto MS medium without antibiotics, and co-cultured in the dark for 3 days, then recollected by nylon net.

Transient transformation of HC and GL3 tissue cultured shoots, followed the protocol according to Yang *et al*. [40]. Healthy young leaves were detached from tissue-cultured shoots and scratched on the abaxial surface underwater then immersed in the *Agrobacterium* resuspension solution. After vacuum treatment for 10 min, the detached leaves were placed on the MS medium with the back facing up and cultured in the dark for 1 day, followed by 2 days of culture under light.

### Transcription factor regulation assay

For GUS activity assays, the recombinant plasmids (pBI121-*MdCWINV1pro* and pCAMBIA-2300-*MdWRKY20*) were cotransformed into callus (as described above) through *Agrobacterium*-mediated transient transformation. GUS enzyme activity was detected using the GUS Reporter Gene Quantitative Detection Kit (Beijing Coolab Technology Co., Ltd.). GUS staining was performed by measuring the production of 4-methylumbelliferyl β-D-glucuronide (4-MU), and the fluorescence intensity (FI) was measured by a Victor Nivo Multimode Plate Reader (PerkinElmer, Waltham, MA, USA) [41].

The ChIP-qPCR assay was performed according to the method described by Wang *et al*. [22]. The BeyoChIP™ ChIP Assay Kit (Protein A/G magnetic beads) was used in accordance with the manufacturer’s instructions. The pCAMBIA-2300-*MdWRKY20* vector was transiently transformed into the leaves of HC and GL3 apple shoots to detect the binding of MdWRKY20 to the *MdCWINV1* promoter *in vivo*. The immunoprecipitation experiment was repeated three times, and the enriched DNA fragments from each reaction were used as a separate biological replicate for qPCR analysis. Nonbinding regions were used as negative controls.

For the dual-luciferase assay, the recombinant plasmids (pGreenII0800-luc-p1/p2/p3/p4/p5/p6/p7/p8 and pGreenII62-sk-*MdWRKY20*) were cotransformed into tobacco leaves using *Agrobacterium* infiltration. The leaves were then kept in the dark for 3 days. Samples were taken for analysis using the Luciferase Reporter Gene Assay Kit (Yeasen Biotechnology, Shanghai, China) according to the instructions.

### Statistical analysis

All data were analyzed using SPSS Statistics 21 (IBM, Armonk, NY, USA) and graphed with SigmaPlot 12.0 (Systat software, Palo Alto, CA, USA) and GraphPad Prism 8 (GraphPad Software, San Diego, CA, USA). The data were analyzed using Student’s *t*-test and one-way analysis of variance (ANOVA) at the significance level of *P* ≤ 0.05. The values are presented as the mean ± standard error (SE) of at least three biological replicates per measurement.

## Supplementary Material

Web_Material_uhaf330

## Data Availability

All relevant data supporting our findings are available in the manuscript and Supplementary Materials. RNA sequencing data have been deposited in NCBI under accession number PRJNA1344609.
