# Supplementary figures and images for: Promoter variation affects binding affinity of the transcription factor MdWRKY20 to the Cell Wall Invertase 1 gene and decreases fructose content in apple fruit
Source: Hortic Res. 2025 Dec 5;13(3):uhaf330. doi: 10.1093/hr/uhaf330 (PMC12966002; doi:10.1093/hr/uhaf330)

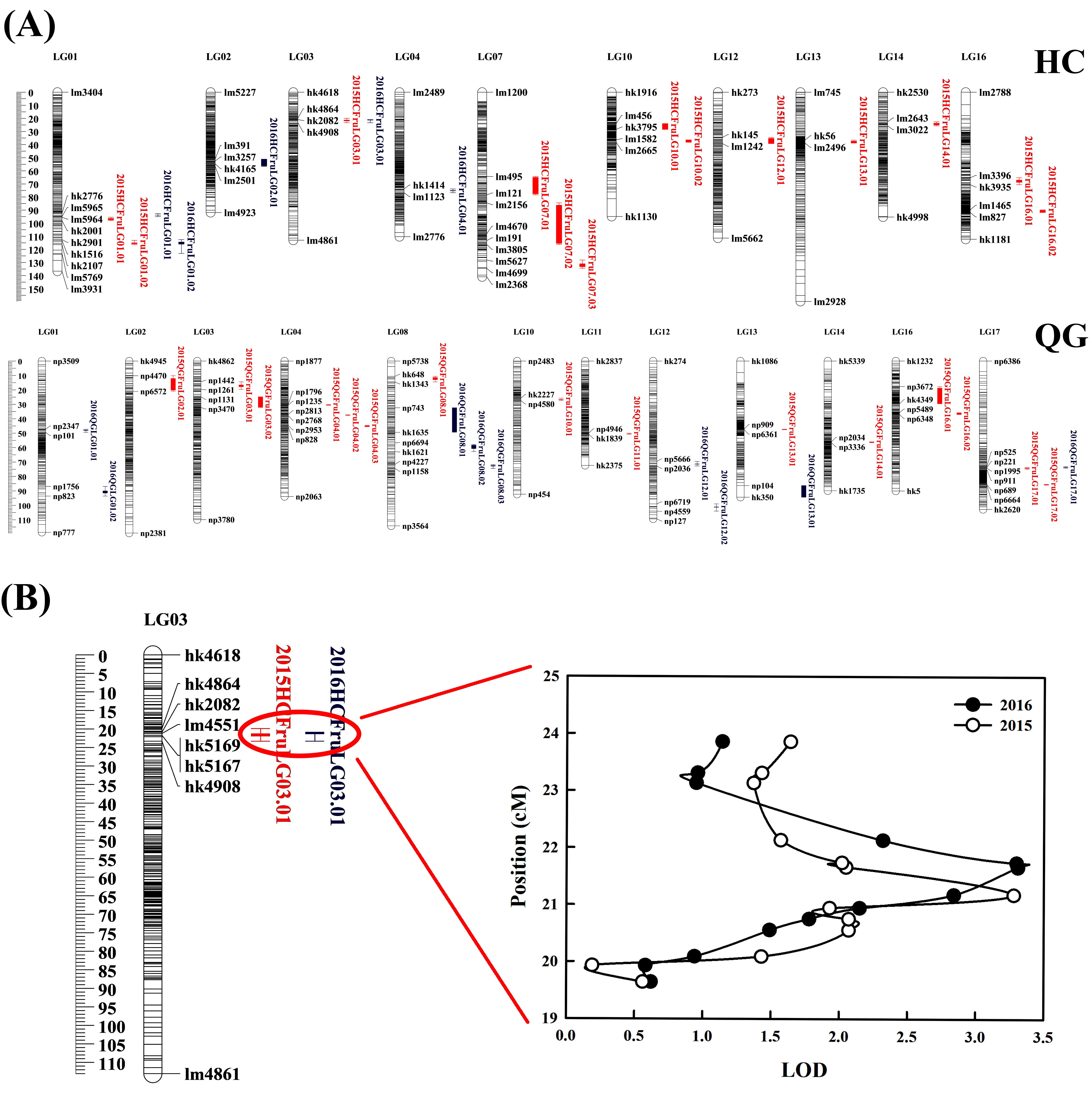

Supplement: Web_Material_uhaf330 [file web_material_uhaf330.zip › Figure S1.jpg]

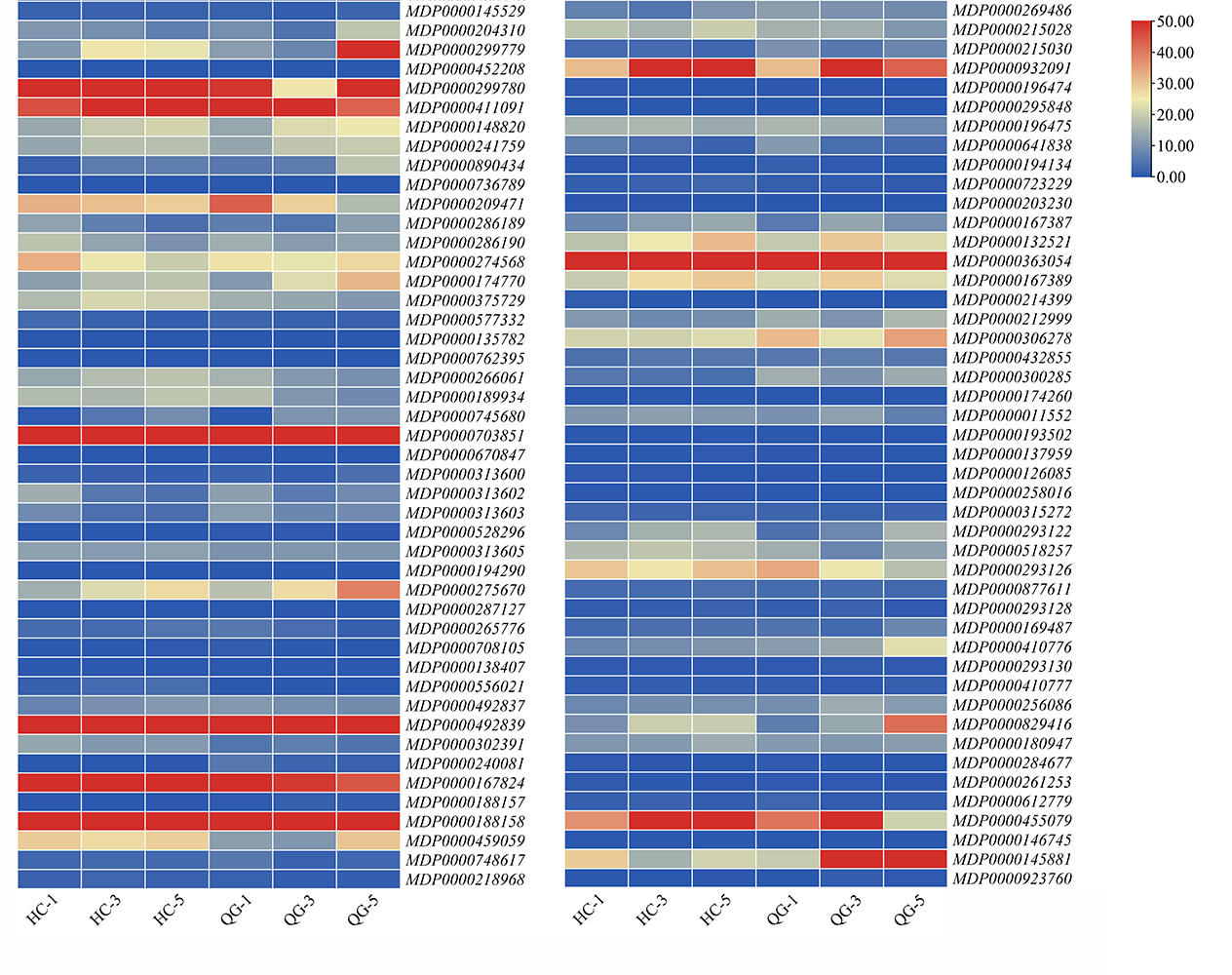

Supplement: Web_Material_uhaf330 [file web_material_uhaf330.zip › Figure S2.jpg]

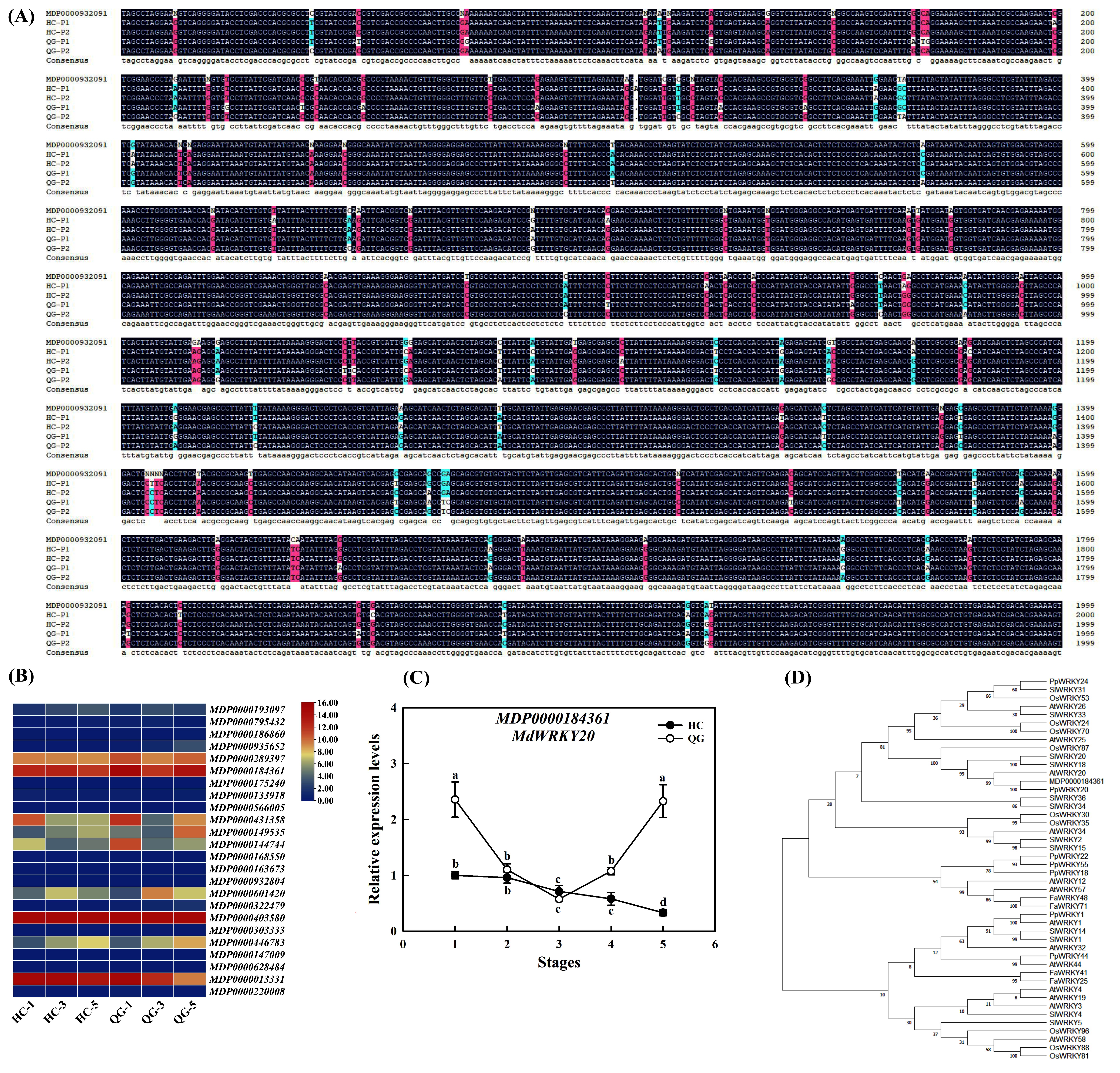

Supplement: Web_Material_uhaf330 [file web_material_uhaf330.zip › Figure S3.jpg]
